# Perfusion index as a predictor for central hypovolemia in humans

**DOI:** 10.1186/cc9489

**Published:** 2011-03-11

**Authors:** A Lima, M Van Genderen, E Klijn, S Bartels, J Van Bommel, J Bakker

**Affiliations:** 1 Erasmus MC University Medical Centre Rotterdam, the Netherlands

## Introduction

In low flow shock, almost 30% of the circulating volume may be lost before hypotension occurs. Thus, shock should be early recognized prior to the development of hypotension. An earlier sign to look for is vasoconstriction in peripheral tissues due to neurohumoral response to the low circulating volume. The perfusion index (PI) derived from the pulse oximetry signal permits a quantitative analysis of variations of peripheral circulation. However, its ability to detect peripheral vasoconstriction due to neurohumoral response in central hypovolemia induced by lower body negative pressure (LBNP) has never been studied.

## Methods

The PI was measured in 24 healthy volunteers during the LBNP test using the pulse oximetry Masimo SET Perfusion Index. The LBNP protocol consisted of 5-minute baseline measurements in the supine position followed by stepwise increases of negative pressure from 0 to -20, -40, -60, -80 and 0 mmHg. HR, BP, and cardiac output were recorded during all of the procedure using a Finometer Blood Pressure Monitor.

## Results

**Figure 1:**
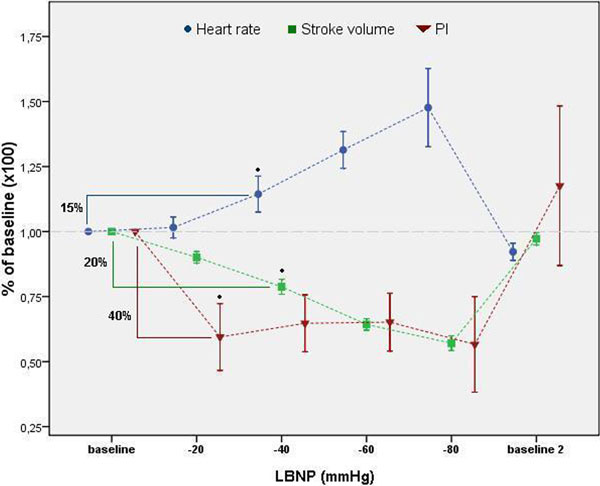
**Correlation between HR, SV and PI**.

Subjects were all male (age mean: 23 ± 6). Figure 1 shows that in all subjects the PI decreased significantly by 40% (*P *= 0.03) during the first -20 mmHg, and kept in this range during the whole experiment. SV decreased significantly by 20% at -40 mmHg. The HR increased significantly by 15% at -40 mmHg. SV and HR changes were proportional to the level of negative pressure in the chamber. No significant changes in BP and CO were observed.

## Conclusions

PI is a sensitive indicator of acute hemodynamic responses to the LBNP-induced central hypovolemia. In addition, it could detect hypovolemia earlier than the 20% decrease in stroke volume.

